# Validity and reliability of the Utrecht Work Engagement Scale-Student Version in Sri Lanka

**DOI:** 10.1186/s13104-018-3388-4

**Published:** 2018-05-04

**Authors:** Nuwan Darshana Wickramasinghe, Devani Sakunthala Dissanayake, Gihan Sajiwa Abeywardena

**Affiliations:** 1 Department of Community Medicine, Faculty of Medicine and Allied Sciences, Rajarata University of Sri Lanka, Saliyapura, 50008 Sri Lanka; 2 Department of Community Medicine, Faculty of Medicine, University of Peradeniya, Peradeniya, 20400 Sri Lanka; 3 Teaching Hospital-Kandy, Kandy, 20000 Sri Lanka

**Keywords:** Work engagement, UWES-S, Collegiate cycle, Sri Lanka, Exploratory factor analysis, Validity, Reliability

## Abstract

**Objective:**

The present study was aimed at assessing the validity and the reliability of the Sinhala version of the Utrecht Work Engagement Scale-Student Version (UWES-S) among collegiate cycle students in Sri Lanka.

**Results:**

The 17-item UWES-S was translated to Sinhala and the judgmental validity was assessed by a multi-disciplinary panel of experts. Construct validity of the UWES-S was appraised by using multi-trait scaling analysis and exploratory factor analysis (EFA) on data obtained from a sample of 194 grade thirteen students in the Kurunegala district, Sri Lanka. Reliability of the UWES-S was assessed by using internal consistency and test–retest reliability. Except for item 13, all other items showed good psychometric properties in judgemental validity, item-convergent validity and item-discriminant validity. EFA using principal component analysis with Oblimin rotation, suggested a three-factor solution (including vigor, dedication and absorption subscales) explaining 65.4% of the total variance for the 16-item UWES-S (with item 13 deleted). All three subscales show high internal consistency with Cronbach’s α coefficient values of 0.867, 0.819, and 0.903 and test–retest reliability was high (p < 0.001). Hence, the Sinhala version of the 16-item UWES-S is a valid and a reliable instrument to assess work engagement among collegiate cycle students in Sri Lanka.

## Introduction

Keeping on par with the emerging trend towards a positive psychology focusing on optimal functioning rather than on malfunctioning, a growing enthusiasm is evident in student engagement research during the last few decades [1].

Work engagement is defined as, “a positive, fulfilling, work-related state of mind that is characterised by vigor (VI), dedication (DE), and absorption (AB). Rather than a momentary and specific state, engagement refers to a more persistent and pervasive affective-cognitive state that is not focused on any particular object, event, individual or behaviour” [2].

Engagement of students in their academic environment has been studied in numerous disciplines [3–10] and the global literature suggests that students’ engagement is positively linked with their academic performances [11, 12] and positive academic outcomes [13–16]. Hence, educators and policymakers are increasingly focusing on student engagement as a means of addressing problems of negative academic outcomes of varying student groups [17].

Amongst the different assessment tools of student work engagement, the most commonly used instrument is the Utrecht Work Engagement Scale-Student Version (UWES-S) [2, 18–22], which is a 17-item self-report measure assessing VI, DE, and AB subscales.

Even though a plethora of research has been conducted on work engagement among varying student populations across the globe, there is a paucity of literature in the South Asian context with no published literature on work engagement among Sri Lankan students, mainly owing to the lack of validated assessment tools. In the context of ever-increasing burden of mental health problems in the Sri Lankan collegiate cycle students [23–26], exploration of effective strategies for mental health and wellbeing promotion has become a timely need. Hence, the present study was designed to assess the validity and the reliability of a culturally adapted Sinhala version of the UWES-S among collegiate cycle students in Sri Lanka.

## Main text

### Methods

#### Structure of the UWES-S

The 17-item UWES-S is a self-administered questionnaire (SAQ) with six, five, and six items assessing VI, DE and AB subscales respectively, measured on a seven-point Likert scale anchored by the response options from 0 (never) to 6 (every day).

#### Translation and pre-testing of the UWES-S

The 17-item UWES-S was translated to Sinhala by using the forward–backward translation method [27–29], involving two independent bilingual translators, who are fluent in Sinhala and English.

The synthesised forward translation of UWES-S was pre-tested among a sample of 25 grade thirteen students outside the study setting. In a subsequent structured interview, the clarity in understanding, acceptability, and comprehension of items and feasibility of using the questionnaire were assessed. None of the items of the questionnaire was claimed to be difficult to understand.

#### Appraising the judgemental validity of the UWES-S

The face, content, and consensual validity were assessed. Using a modified Delphi technique, a multi-disciplinary panel of experts in the fields of psychiatry, psychology, public health, teaching, student counseling, and medical education has assessed each item on its relevance, appropriateness, and acceptability in the local context for assessing burnout among collegiate cycle students based on a rating scale from 0 (strong disagreement) to 10 (strong agreement). At the end of this iterative process, except for the item 13 stating, “When studying, I am very resilient, mentally”, all other items had a median score more than 7 for all the aspects. Thus, it was decided to include all 17 items for the assessment of construct validity.

#### Study design and setting

This school-based, cross-sectional validation study was conducted in the Kurunegala district, North Western province, Sri Lanka from May 2014 to April 2015. In Sri Lanka, the collegiate cycle in the education system consists of grade twelve and thirteen. Three Sinhala medium government schools in the Kurunegala district having all four collegiate cycle subject streams, viz., Science, Arts, Commerce and Technology were selected.

#### Study participants

From the selected schools, three grade thirteen classes each were selected representing both male and female students studying in all four subject streams. A total of 194 students participated in the study in their classrooms and each participant filled the SAQ independently. The response rate was 100.0% and 55.2% (n = 107) of the sample were females. The mean age was 18.3 years (SD = 0.43 years). The majority of students were studying in the Science stream (n = 78, 40.2%). The numbers of students in the Arts, Commerce and the Technology streams were 60 (30.9%), 41 (21.2%), and 15 (7.7%) respectively.

#### Data analysis

Data were analysed by using the Statistical Package for Social Sciences (SPSS) version 17.0. Preparatory data analysis showed that there were no violations of the assumptions related to the data analytic techniques. Given that the participants to variables ratio was 11.4, the sample size was adequate for factor analysis [30]. Even though there were few items that showed non-normal distribution of data, it is considered to be a ubiquitous phenomenon in psychological assessment research.

#### Multi-trait scaling analysis

Item-scale correlations were analysed and item-convergent and item-discriminant validity were assessed. A stringent criterion of correlation of 0.40 or greater between an item and its own subscale was considered as a success for assessing item-convergent validity. Furthermore, in assessing item-discriminant validity, items that correlated significantly higher (more than 1.96 standard errors) with its own subscale than with the other two subscales, were considered as scaling successes.

#### Exploratory factor analysis (EFA)

EFA was conducted by Principal Component Analysis (PCA) with Oblimin rotation. Kaiser’s criterion/eigenvalue, scree plot and parallel analysis were used to decide the number of factors to retain. The factors that lead to a meaningful interpretation and theoretical sense were ultimately selected.

#### Assessment of reliability

Reliability was assessed using internal consistency and test–retest reliability by re-administering the SAQ to a sub sample of 22 grade thirteen students after 2 weeks.

## Results

### Descriptive statistics of the UWES-S scores

Descriptive statistics of UWES-S subscales are given in Table 1. The mean item score was highest in the AB subscale (4.52, SD = 1.40) and was lowest in the DE subscale (3.70, SD = 1.23).

**Table 1:** Descriptive statistics of the UWES-S subscale scores among grade thirteen students (n = 194)

Subscale	Mean total score	SD	Mean item score	SD
VI	22.66	6.34	3.78	1.06
DE	18.48	6.14	3.70	1.23
AB	27.14	8.43	4.52	1.40

### Multi-trait scaling analysis

Table 2 summarises the results of the multi-trait scaling analysis and as per the predetermined cut-off values, except for item 13, item-convergent validity and item-discriminant validity were confirmed for other 16 items in the UWES-S.

**Table 2:** Analysis of item-scale correlations to determine item-discriminant scaling successes for the UWES-S scores (n = 194)

Code	Item	VI score	DE score	AB score	Standard error	Cut-off value (− 1.96 SE)	Scaling success
VI1	When I study, I feel like I am bursting with energy	*0.824*	0.357	0.344	0.041	0.744	Success
VI4	When studying I feel strong and vigorous	*0.835*	0.323	0.379	0.039	0.757	Success
VI7	When I get up in the morning, I feel like going to school	*0.834*	0.453	0.419	0.039	0.756	Success
VI10	I can continue for a very long time when I am studying	*0.661*	0.088	0.166	0.054	0.554	Success
VI13	When studying, I am very resilient, mentally	0.046	0.224	0.089	0.070	0.086	Not success
VI16	When studying I always persevere, even when things don’t go well	*0.698*	0.467	0.453	0.052	0.596	Success
DE2	I find my studies to be full of meaning and purpose	0.468	*0.774*	0.514	0.046	0.684	Success
DE5	My studies inspire me	0.447	*0.762*	0.500	0.047	0.670	Success
DE8	I am enthusiastic about my studies	0.356	*0.781*	0.493	0.045	0.692	Success
DE11	I am proud of my studies	0.358	*0.709*	0.502	0.051	0.609	Success
DE14	I find my studies challenging	0.324	*0.790*	0.556	0.044	0.703	Success
AB3	Time flies when I’m studying	0.472	0.628	*0.861*	0.037	0.789	Success
AB6	When I am studying, I forget everything else around me	0.457	0.608	*0.858*	0.037	0.785	Success
AB9	I feel happy when I am studying intensively	0.333	0.523	*0.797*	0.043	0.711	Success
AB12	I am immersed in my studies	0.350	0.527	*0.841*	0.039	0.764	Success
AB15	I can get carried away by my studies	0.379	0.541	*0.838*	0.039	0.761	Success
AB17	It is difficult to detach myself from my studies	0.353	0.467	*0.718*	0.050	0.619	Success

### Exploratory factor analysis

Item 13 was found to have poor psychometric properties in judgemental validity, item-convergent validity, and item-discriminant validity. Deletion of item 13 from the subscale also improved the internal consistency. Hence, both 17-item UWES-S and 16 items of the UWES-S (item 13 deleted) were subjected to PCA. The Kaiser–Meyer–Olkin Measure were 0.851 and 0.855 respectively and Bartlett’s test of sphericity reached statistical significance (p < 0.001) for both versions.

Only three factors had eigenvalues more than 1.0, which cumulatively explained 63.9% and 65.5% of the total variance for the 17-item and 16-item UWES-S versions respectively. For both versions, the parallel analysis showed only two factors with eigenvalues exceeding the corresponding criterion values and the screeplot showed a clear break after the second factor.

However, for both versions, the component matrix in PCA with unrotated loadings revealed a number of items of UWES-S loading on the third factor with values greater than 0.3. Furthermore, the pattern matrix generated in PCA using direct Oblimin rotation revealed that seven items had loading values more than 0.3. Based on this collective evidence, it was decided to ‘force’ a three-factor solution for further investigation.

Even though both Varimax rotation and Oblimin rotation was used, it was decided to report statistics related to Oblimin rotation as the factors were strongly correlated (> 0.3). The rotated three-factor solution revealed a simple structure with all three factors showing a number of strong loadings. In the 17-item UWES-S version, the three-factor solution explained a total of 63.9% of the variance, with factor one contributing 41.4%, factor two contributing 14.9%, and factor three contributing 7.6%, whereas, the corresponding values for the 16-item UWES-S version were 65.4, 44.0, 14.2, and 7.2%. Table 3 shows the pattern and structure matrix for PCA with Oblimin rotation of three-factor solution of 16-item UWES-S.

**Table 3:** Pattern and structure matrix for PCA with Oblimin rotation of three-factor solution of 16-item UWES-S

Item	Pattern coefficients			Structure coefficients			Communalities
	Factor			Factor			
	1	2	3	1	2	3	
AB3	*0.748*	0.066	0.127	*0.848*	0.357	0.606	0.734
AB6	*0.766*	0.062	0.100	*0.848*	0.351	0.588	0.729
AB9	*0.834*	− 0.051	− 0.012	*0.810*	0.228	0.485	0.659
AB12	*0.869*	0.002	− 0.043	*0.843*	0.283	0.491	0.712
AB15	*0.862*	0.004	− 0.034	*0.843*	0.286	0.496	0.711
AB17	*0.718*	0.007	− 0.009	*0.715*	0.247	0.434	0.511
VI1	− 0.038	*0.838*	0.109	0.313	*0.858*	0.340	0.744
VI4	0.080	*0.858*	− 0.016	0.361	*0.881*	0.292	0.780
VI7	− 0.017	*0.693*	0.288	0.394	*0.774*	0.486	0.669
VI10	0.023	*0.905*	− 0.268	0.165	*0.832*	0.020	0.751
VI16	0.136	*0.539*	0.227	0.458	*0.653*	0.473	0.521
DE2	-0.036	0.125	*0.788*	0.490	0.351	*0.804*	0.660
DE5	0.011	0.131	*0.688*	0.477	0.343	*0.734*	0.556
DE8	0.004	− 0.085	*0.812*	0.473	0.162	*0.789*	0.628
DE11	0.140	0.035	*0.587*	0.512	0.260	*0.683*	0.482
DE14	0.161	− 0.120	*0.709*	0.556	0.149	*0.772*	0.619

The interpretation of three-factor solution of the UWES-S with item 13 deleted, was consistent with previous research on the UWES-S, with subscale items of VI, DE, and AB loading strongly on three different factors. Hence, the three-factor solution with item 13 deleted was considered as the validated Sinhala version of the UWES-S.

### Assessment of reliability

The impact of each item on the related subscale was assessed by computing Cronbach’s α when the respective item is deleted. None of the items in DE subscale and AB subscale improved the overall Cronbach’s α value; however, deletion of item 13 improved the Cronbach’s α value from 0.696 to 0.867. Hence, item 13 was removed from the UWES-S and the reliability was re-assessed for internal consistency and all three subscales show high internal consistency with Cronbach’s α coefficient values of 0.867, 0.819, and 0.903 for VI, DE, and AB subscales respectively.

There were strong, positive statistically significant (p < 0.001) correlations for each of the three subscales of the UWES-S in test–retest reliability assessment. The correlation coefficients were 0.831, 0.866 and 0.839 for the VI, DE, and AB subscales respectively.

## Discussion

The present study was designed to validate the Sinhala version of the UWES-S among Sri Lankan collegiate cycle students addressing an important research vacuum on work engagement research in high school students in the South Asian context. A cross-sectional study design and the sample size of the study deemed appropriate with the study objective.

In assessing the judgemental validity, item 13 (“When studying, I am very resilient, mentally”) had the least median rating score and the main concern raised regarding the item was the difficulty in explaining the phrase “mentally resilience” in Sinhala language. The global literature suggests that modified versions of the UWES-S with removal of several items have been used in work engagement among high school, college, and university students [21, 22, 31].

Even though the three-factor structure of the Utrecht Work Engagement Scale (UWES) has been confirmed in a large number of studies [32–36], the limited number of studies that explored the factor validity of the UWES-S have failed to provide convincing evidence to support the three-factor structure of the UWES-S [37]. Furthermore, the findings of the study conducted by Sonnentag [38], raises concerns about the three-factor structure of UWES. This evidence coupled with the fact that UWES-S is a relatively novel measure in the South Asian context, EFA was employed to assess the construct validity, instead of using confirmatory factor analysis [39].

The three-factor solution of the original 17-item UWES-S, explained 63.9% of the variance, whereas the three-factor solution of the UWES-S with item 13 deleted, explained 65.4% of the variance. Furthermore, the interpretation of the three-factor solution of the UWES-S with item 13 deleted, was consistent with previous research on the UWES-S with subscale items of VI, DE, and AB loading strongly on three different factors [2, 22].

In assessing the internal consistency, deletion of item 13 improved the Cronbach’s α value from 0.696 to 0.867 and all three subscales showed high internal consistency. This finding is consistent with findings of studies using the Portuguese version [22], Spanish version [22], Dutch version [2, 22], Turkish version [21] and Romanian version [20] of the UWES-S.

The present study also revealed that there were statistically significant strong positive correlations for each of the three subscales of UWES-S in the test–retest reliability assessment. Though these findings are consistent with the previous study findings [21], the values were not as high as those reported in that study.

In conclusion, the study findings indicate that the Sinhala version of the 16-item UWES-S is a valid and a reliable instrument to assess work engagement among collegiate cycle grade thirteen students in Sri Lanka. Due to its brevity, ease of administration and sound psychometric properties, it could be used as an effective screening tool at the school level. The study findings pave the way to establish the initial evidence base for the relevance and the applicability of the concept of work engagement in the South Asian context.

## Limitations

This study has some limitations. Given that the study sample was recruited not using a probability sampling technique in a selected district of Sri Lanka, the generalisability of the study findings to other populations should be done with caution, considering the variations in educational and cultural contexts. Furthermore, given that the three-factor structure of the UWES-S is established in the present study, confirmatory factor analysis procedures are recommended for future studies.

## Abbreviations

AB: absorption
DE: dedication
EFA: exploratory factor analysis
PCA: principal component analysis
SPSS: Statistical Package for Social Sciences
UWES-S: Utrecht Work Engagement Scale-Student Version
VI: vigor
